# GDNF protects enteric glia from apoptosis: evidence for an autocrine loop

**DOI:** 10.1186/1471-230X-12-6

**Published:** 2012-01-17

**Authors:** Martin Steinkamp, Heike Gundel, Nadine Schulte, Ulrike Spaniol, Carolin Pflueger, Eugen Zizer, Georg BT von Boyen

**Affiliations:** 1 Department of Gastroenterology, Endocrinology and Metabolism, University of Gießen und Marburg GmbH, Site Marburg, Baldingerstraße, 35037 Marburg, Germany; 2 Department of Internal Medicine I (Gastroenterology), University of Ulm, Albert-Einstein-Allee 23, 89081 Ulm, Germany

**Keywords:** enteric glia, GDNF, Crohn's disease, IBD, apoptosis

## Abstract

**Background:**

Enteric glia cells (EGC) play an important role in the maintenance of intestinal mucosa integrity. During the course of acute Crohn's disease (CD), mucosal EGC progressively undergo apoptosis, though the mechanisms are largely unknown. We investigated the role of Glial-derived neurotrophic factor (GDNF) in the regulation of EGC apoptosis.

**Methods:**

GDNF expression and EGC apoptosis were determined by immunofluorescence using specimen from CD patients. In primary rat EGC cultures, GDNF receptors were assessed by western blot and indirect immunofluorescence microscopy. Apoptosis in cultured EGC was induced by TNF-α and IFN-γ, and the influence of GDNF on apoptosis was measured upon addition of GDNF or neutralizing anti-GDNF antibody.

**Results:**

Increased GDNF expression and Caspase 3/7 activities were detected in in specimen of CD patients but not in healthy controls. Moreover, inactivation of GDNF sensitized in EGC cell to IFN-γ/TNF-α induced apoptosis.

**Conclusions:**

This study proposes the existence of an autocrine anti-apoptotic loop in EGC cells which is operative in Crohn's disease and dependent of GDNF. Alterations in this novel EGC self-protecting mechanism could lead to a higher susceptibility towards apoptosis and thus contribute to disruption of the mucosal integrity and severity of inflammation in CD.

## Background

In inflammatory bowel disease (IBD), the impairment of the bowel luminal barrier function is suggested as an important pathophysiologic mechanism that triggers inflammatory activity of the mucosa [1-3]. The translocation of luminal antigens into the mucosa perpetuates the inflammatory response, and may contribute to the chronification of the disease [1-5]. Therefore, the integrity of the epithelial lining is essential for gut mucosal homeostasis and integrity [6,7], and cytokines that promote antiapoptotic effects on intestinal epithelial cells or stimulate their proliferation are suggested to be important protective factors. Neurotrophins has been identified as antiapoptotic substances for the colonic epithelial cells [8,9]. The source of neurotrophins and neurotrophic factors could be identified as the enteric glia cells (EGC) of the mucosal plexus [10]. On the other hand, it was shown that in Crohn's disease (CD) the amount of glial cells is reduced [11,12]. This may due to apoptotic events in the EGC, followed by a reduced ability for maintenance of an intact mucosal barrier function. Until now, the exact mechanisms of interaction in this protective network remain unclear.

To date, the EGC seems to represent an ownstanding entity of cell population, since they share some cell properties with astroglia in the central nervous system (CNS), but on the other hand they also exert also similarities to microglia [6,7,13].

In this paper, we aimed to investigate the role of glial-derived neurotrophic factor (GDNF) in the regulation of EGC apoptosis.

## Methods

### Human tissue

The whole research work is conformed to the Helsinki Declaration. The patients enrolled in the study gave their informed consent and the study was approved by the local ethical committee of the University of Ulm, which is leaded by Prof. Dr. U. Brückner. The diagnosis of CD was established by using usual criteria [14]. Inflamed colonic biopsies were taken from 10 patients with CD (5 female/5 male; mean age 34 years; range 23 to 48 years). Biopsies were taken during colonoscopy. The mean duration of CD was 4.5 years. No patients received biologics. 4 CD patients were treated with azathioprine, one patient with 6-MP and 3 patients with budesonide. The other patients were not treated at the time of study and had only less clinical signs of activity.

As controls healthy colonic biopsies were taken from 26 patients, which underwent a routine screening colonoscopy (1 female/4 male; mean age 56 years; range 51 to 60 years). Tissue GFAP, GDNF and c-Caspase-3 levels were measured by immunofluorescence. Patient informed consent for taking and analysis of biopsy specimen was obtained. The study was approved by the local ethics comittee.

### Indirect immunofluorescence

Tissue-biopsies were deparaffinized and permeabilized with PBS/0.3% Triton X100. Antigen retrieval was performed by boiling the slides in 0.01 M trisodium citrate buffer, pH 6, for 10 min. Sections were then preincubated with 10% normal goat serum containing 0.2% Triton X-100 overnight at 4°C to block nonspecific binding. Slides were then incubated over night at 4°C with Antibodies against GFAP (Pharmingen, San Diego, USA, mouse, 1:100), GDNF (Abcam, USA, rabbit, 1:100) or GDNF (Sigma-Aldrich, USA, mouse, 1:80) and rabbit anticleaved caspase-3 (1:200, Cell Signalling Technology).

After washing in PBS/0,1% Tween 20, the slides were incubated with the appropriate secondary antibodies: cy3 coupled goat anti-mouse IgG (DPC Biermann, Bad Nauheim, Germany) or cy3 coupled goat anti-rabbit IgG (DPC Biermann, Bad Nauheim, Germany) and Alexa 488 coupled goat anti-rabbit IgG (Sigma) or Alexa coupled goat anti-mouse IgG (Sigma). Dilutions of the secondary antibodies were 1:800. After washing in PBS, slides were embedded in glycerol gelatine. Control labelling was performed with omission of the first antibodies to ensure that there was no unspecific labelling of cells.

Tissue-biopsies were analyzed using a Leica confocal laser-scanning microscope. Single optical sections were recorded under conditions, which exclude cross-activation of the individual signal channels.

### Histological analysis

Every three Immunolabelled biopsies of 10 CD and 5 control persons were used for evaluating the expression of GFAP, GDNF and cCaspase-3 in EGCs.

### Dissociated EGC cultures

Newborn rats (Wistar strain) were decapitated. The intestines were removed and the myenteric plexus was isolated as previously described (21). In brief, small intestines were rinsed in sterile MEM with 25 mM HEPES buffer (Gibco Life Technologies, California USA). The muscle layer containing the myenteric plexus was stripped from the mucosa. The tissue was incubated in a collagenase solution (CL type II, Gibco, 1 mg/ml) in Hanks balanced salt solution (Gibco) for 1.5 h at 37°C. The disintegrated tissue was vortexed and the isolated parts of the myenteric plexus were stored in MEM on ice. The collected pieces were incubated in trypsin (0.1 mg/ml, Gibco) for 15 min at 37°C and then centrifuged. Trypsinisation was stopped by addition of fetal calf serum (Gibco). Then cells were plated on polyornithine coated (0.5 mg/ml, Sigma, Schnelldorf, Germany) coverslips and were topped with 450 μl DMEM-F-12 (Gibco). The cultures were kept in a humidified atmosphere of 95% air/5%CO_2 _at 37°C. At day 3, the culture consisted of approximately 98% of EGCs.

### Indirect immunofluorescence

The EGCs of each animal were pooled and cultured on 20 different coverslips. Cell cultures were fixed and permeabilized with 90% methanol, 10% acetic acid cooled down to -40°C. After washing in PBS cells were blocked with 1% bovine serum (Sigma) for 40 min, anti-GFR-α1-3 and Ret (Becton Dickinson Transduction, Germany, R&D Systems, Germany, Santa Cruz, Germany) antibodies were incubated for 1 h at room temperature. Immunofluorescence staining was done by secondary antibodies: goat anti-mouse IgG (cy3, Sigma) or rabbit anti-goat (cy3, Becton Dickinson, Transduction, Germany). After immunostaining, coverslips were mounted cell side down on microscope slides using moviol. Cell cultures were analyzed using a Leica confocal laser-scanning microscope.

### Apoptosis in EGC cultures induced by tumor necrosis factor-α and interferon-γ

Rat primary enteric glia was isolated as described above. For induction of apoptosis, the glia was seeded in 48 well plates. After reaching confluence, the cells were washed twice with PBS, and thereafter 180 μl Dulbecco's minimal essential medium (DMEM) was added, containing 100 ng/ml interferon-γ, 100 ng/ml TNF-α, both or carrier. Then, the cultures were incubated with or without GDNF 100 ng/ml, or carrier over a period of 40 hours. In case of the addition of neutralizing antibody against GDNF, the cells were preincubated for 1 hour with anti-GDNF 0,5 μg/ml. For detection of caspase 3/7 activity in the ECG cultures, the caspase substrate Rhodamin 110 was added in the relation 1:1 to the wells according to the instructions of the manufacturer of the assay (ApoOne Homogenous caspase 3/7 Assay, Promega, Germany), and the wells were vortexed at room temperature over 2 minutes at 400 rpm. After additional 2 hours at room temperature, the wells were vortexed again, followed by fluorometric measurement of the caspase 3/7 activity according to the manufacturers recommendations. In brief, the stimulation wavelength was 485/20, the emission wavelength was 528/20. Three independent experiments were performed in duplicates, the results are indicated as mean values ± SD.

### Western blot analysis of GDNF receptors in primary enteric rat glia

Western blot analysis was performed according to standard protocols as described elsewhere [12]. In brief, primary enteric rat glia was seeded in 10 cm diameter culture dishes, and after reaching confluence rinsed with PBS three times. Thereafter, the cells were lysed and prepared as described before [12]. The antibodies for the detection of GFR-α1-3 and Ret was purchased from R&D Systems, Germany. For western blotting, it was used at a concentration of 1:25.

### Data analysis

All data given in the text and figures are expressed as mean values ± SEM. The data were analyzed using non-parametric two-tailed Mann-Whitney U test with p ≤ 0.05 considered as an indicator of significance.

## Results

### GDNF regulation and caspase 3 activation in EGC of patients with CD

**Figure 1:**
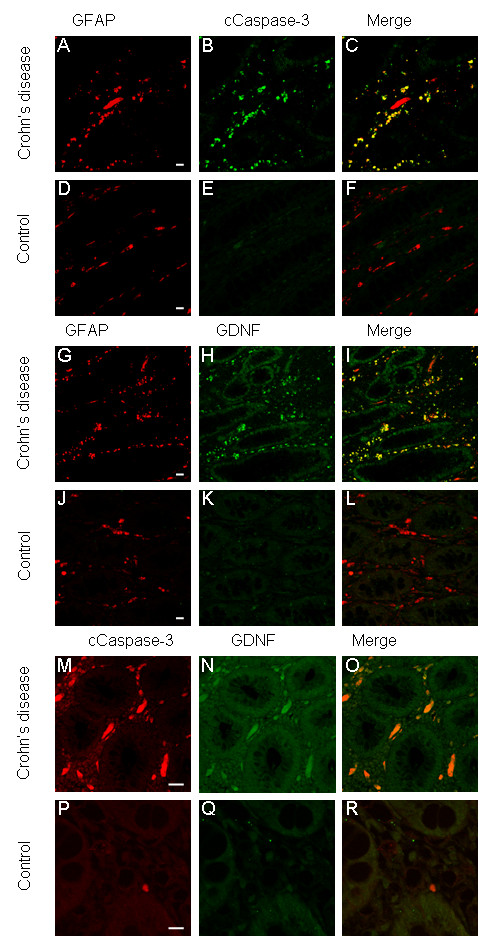
**Biopsies of the inflamed colon of patients suffering from CD and controls were double immunolabelled with anti GFAP (A,D,G,J red) and cCaspase-3 (B,E green) or anti GDNF (H,K green) antibodies and were analyzed by optical sectioning using a confocal microscope**. Both antigens, GFAP (A) and c-Caspase-3 (B) can be detected highly in the intestinal wall of the inflamed colon of CD (A,B). The merged images (C) reveal an almost complete overlap of both immunoreactivities (yellow). Only few GFAP-positive cells (D) display no cCaspase-3 immunoreactivity (E) in the control section (F). Although a high immunoreactivity of GDNF (H) in GFAP-positive cells (G,I) and in the epithelial cells of biopsies of patients with CD can be detected, in control biopsies GFAP-positive EGCs (J), which are positioned in the mucosal plexus in close vicinity to the epithelium of the colon, show no GDNF secretion (K,L). Furthermore the subepithelial cells, which express GDNF highly (N, green) showed a colocalization with cCaspase-3 in sections of CD (M-O), whereas in controls no relevant apotosis, proofed by cCaspase-3, or GDNF expression could be detected (P-R). Scale bars, 50 um.

In biopsy specimen of patients suffering from CD, caspase 3 activation could be detected in the mucosal glial plexus, and this was coincident to GFAP and to GDNF expression in the same structures. In controls, neither caspase 3 activation nor GFAP or GNDF upregulation was recognized (Figure 1). These results suggest the occurence of apoptosis in the mucosal plexus and GDNF upregulation in the context of chronic bowel inflammation.

### GDNF receptor expression in rat primary EGC cultures

**Figure 2:**
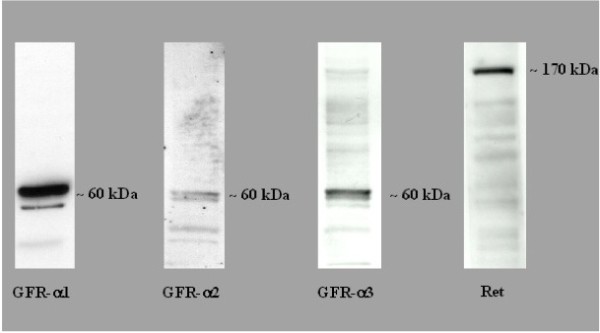
**Detection of the receptors GFR-*α *1-3 and Ret receptor by Western Blot**. The molecular weight (kDa) of the protein are shown on the right of the western blot figures. These blots are representative of 3 independent experiments (N = 3).

**Figure 3:**
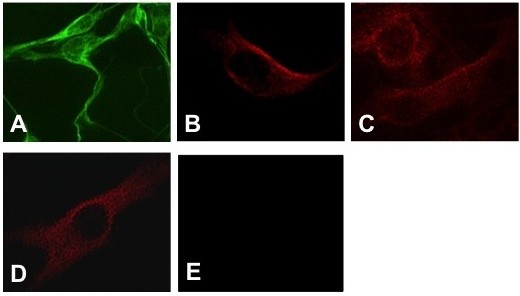
**Detection of GFR-*α *1 (A) -2 (B) -3 (C) and Ret receptor (D) in primary EGC culture by Indirect immunofluorescence**. (E) Negative control under omission of first antibody. Magnification: 20fold. These stains are representative of 3 independent experiments (N = 3).

The expression of the GDNF receptors GFR-α1, GFR-α2, GFR-α3 and the coreceptor Ret was determined from lysates of EGC cultures using western blot. We were able to detect specific bands for all GDNF receptors (Figure 2). Furthermore, we performed an indirect immunofluorescence staining of EG cells cultures to prove these results in a second method (Figure 3). We confirmed our results, as all receptors were specifically detectable in this second method. The blots are presented to confirm the presence of the receptors, but not the quantity or different expression levels. Therefore, a GAPDH control is omitted.

### TNF-α and IFN-γ induces apoptosis in EGC, influence of GDNF and anti-GDNF

**Figure 4:**
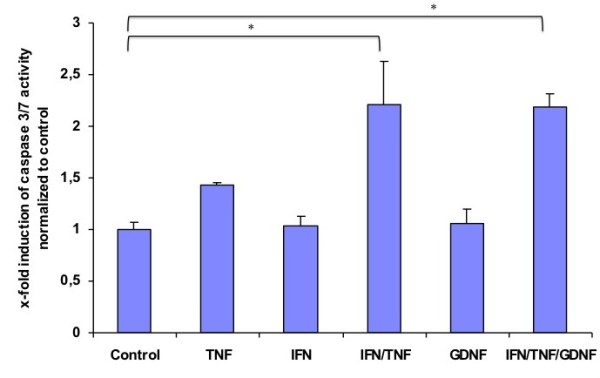
**Activation of caspase 3/7 in primary EGC cultures**. Fluorometric detection of caspase 3/7 activity after incubation of the EGC cultures with TNF-α (100 ng/ml), IFN-γ (100 ng/ml) and GDNF (100 ng/ml), alone or in combination as described above. The bars indicate mean values ± SD, normalized to controls, three independent experiments were performed in duplicates (N = 3). Asterisk indicates a significant increase as compared to controls (p < 0.05).

**Figure 5:**
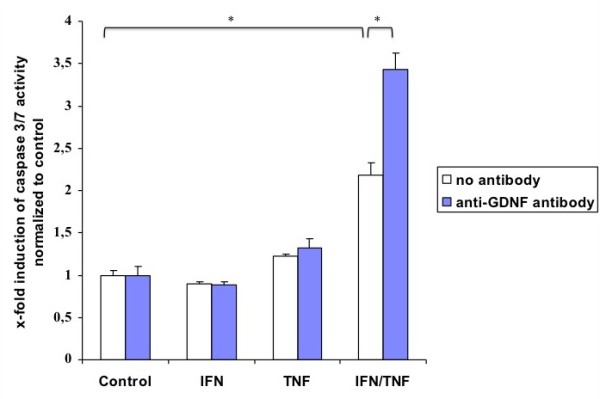
**Activation of caspase 3/7 activity in primary EGC cultures**. Fluorometric detection of caspase 3/7 activity after incubation of the EGC cultures with TNF-α (100 ng/ml), IFN-γ (100 ng/ml), neutralizing anti-GDNF-antibody (0.5 μg/ml), alone or in combination as described above. The bars indicate the mean values ± SD, normalized to controls, three independent experiments were performed in duplicates (N = 3). Asterisk indicates a significant difference between both values (p < 0.05).

Since glial cells are assumed as highly apoptosis resistant cells, we aimed to expose these cells to an inflammatory cytokine environment using TNF-α and IFN-γ. Neither of these factors alone was able to induce apoptosis in primary rat EGC cultures, but in combination they significantly increased apoptosis rates in the cultures by nearly 2 fold, as indicated by a caspase 3/7 activation in these cells. Noteworthy, we have also tested various other combinations of important cytokines with implication in Crohn's disease (e.g. IL-1ß, IL-6) but failed to induce apoptosis (data not shown). The addition of GDNF had no effect on the apoptosis rate in the cell cultures, but it was also not able to alter the apoptosis rate in the cultures after induction of apoptosis by the combination of TNF-α and IFN-γ (Figure 4). However, if a neutralizing antibody against GDNF was added to neutralize endogenous secreted GDNF, the apoptosis rates in the cultures that were stimulated with TNF-α and IFN-γ raised significantly (Figure 5). Therefore, it is likely that an autocrine antiapoptotic loop is activated in EGC when exposed to inflammatory conditions.

## Discussion

Recent molecular and biochemical analysis as well as cell biology approaches demonstrated important regulatory functions of mucosal EGC in the assembly and maintenance of intestinal mucosa integrity [9,10]. This is underscored by the potential of EGC to secrete key cytokines and growth factors such as interleukin-6 (IL-6), and TGF-ß1 [15,16], which ensure cell communication with the microenvironment. EGC thereby influence local immune responses and control vital cellular functions including differentiation, growth and survival. Consistent with the capacity of EGC cells to control survival and cell death, we have most recently shown that GDNF, when expressed and secreted from primary rat enteric glia cells, can protect epithelial cells from apoptosis [9]. It is therefore not surprising that reduced EGC levels, as frequently found in active Crohn's disease or following different experimental settings in mice, result in disrupted mucosa integrity with increased inflammation and acquisition of hemorrhagic lesions [11,17]. Interestingly, however, the underlying molecular mechanisms of how EGC protect themselves from cytokine mediated apoptosis remained elusive.

Here, we addressed this issue and identified a novel regulatory loop which allows EGC cells to escape from cell death. We show for the first time that GDNF, which is produced and secreted by EGC cells feeds back in an autocrine manner to protect EGC from apoptosis, thus contributing to integrity of the mucosal EGC plexus.

We have identified high level of GDNF expression in mucosal EGC from patients with Crohn's disease, whereas GDNF was not detectable in healthy controls. Interestingly, our analysis further revealed remarkable expression of all subclasses of GDNF receptors, namely GFR-α1-3, and the coreceptor Ret in primary EGCs, indicating that EGC themselves may be target cells for secreted GDNF under inflammatory conditions. To test this hypothesis, we decided to challenge EGC cultures with TNF-α and IFN-γ, two major apoptosis related cytokines whith key functions in Crohn's disease. Neither TNF-α nor IFN-γ alone caused a significant effect on EGC cultures with respect to cell death, thus supporting a previously suspected relative insensitivity of EGC to apoptosis. However when both cytokines were combined, we observed a significant increase in apoptosis, as measured by caspase 3/7 activation. Even more important, however, induction of apoptosis by TNF-α/IFN-γ was further and most dramatically increased upon inactivation of GDNF. These findings not only underscore the relevance of GDNF in regulation of EGC cells but also emphasize a so far unknown cell protective and anti-apoptotic function.

Together with our aforementioned finding that EGC express all receptors for GDNF and in addition, produce high amounts of GDNF in bowel inflammation [10], these results allow us to hypothesize the existence of an autocrine loop that prevents EGC from apoptosis. Moreover, disruption of this novel regulatory mechanism by addition of a neutralizing antibody against GDNF uncovered strong antiapoptotic effects of GDNF, which might have important implications in the preservation and maintenance of mucosal glia integrity and apoptosis resistance.

## Conclusions

Therefore, we conclude that GDNF is an important endogenous factor for the regulation of EGC apoptosis, and is part of a network of protective substances that may be important for mucosal integrity and healing. Furthermore, alterations leading to disruption of this protective network of neurotrophins and neurotrophic factors might contribute to a more severe course of inflammation. The effects shown in this study focus on Crohn's disease. Additional studies on ulcerative colitis are required in future approaches in order to transfer our results to IBD in general.

## Competing interests

The authors declare that they have no competing interests.

## Authors' contributions

GvB and MS treated the patients in the IBD outpatient clinic and performed the colonoscopies. MS and GvB drafted the manuscript. GvB, MS, EZ and HG prepare the EGC cultures and were responsible for the experiments in vitro. NS, CP and HG performed with GvB the microscopically analysis. MS and HG carried out the western blot analysis, indirect immunofluorescence and ELISA. All authors participated in the design of the study. All authors read and approved the final manuscript.

## Pre-publication history

The pre-publication history for this paper can be accessed here:

http://www.biomedcentral.com/1471-230X/12/6/prepub
